# Sexual trauma and post-traumatic stress among African female refugees and migrants in South Africa

**DOI:** 10.4102/sajpsychiatry.v24.i0.1208

**Published:** 2018-06-07

**Authors:** Mpho D. Mhlongo, Andrew Tomita, Lindokuhle Thela, Varsha Maharaj, Jonathan K. Burns

**Affiliations:** 1 Department of Psychiatry, Nelson R Mandela School of Medicine, University of KwaZulu-Natal, South Africa; 2 KwaZulu-Natal Research Innovation and Sequencing Platform (KRISP), College of Health Sciences, University of KwaZulu-Natal, Durban, South Africa; 3 Centre for Rural Health, School of Nursing and Public Health, University of KwaZulu-Natal, Durban, South Africa; 4 Institute of Health Research, University of Exeter, United Kingdom

## Abstract

**Background:**

While there is considerable research in developed countries on the nature and extent of post-traumatic stress among refugees and migrants, few report on female Africans migrating within Africa.

**Aim:**

The aim of this study was to investigate the association between exposure to traumatic life events and post-traumatic stress disorder risk in refugees and migrants in Durban, South Africa, with specific focus on sexual trauma events among women.

**Methods:**

Interviews were conducted on 157 consenting non-South African adults using a sociodemographic questionnaire, Life Events Checklist (documenting traumatic events experienced) and the Harvard Trauma Questionnaire (measuring post-traumatic symptomatology). Associations between total number of traumatic events and post-traumatic stress were explored using adjusted regression models.

**Results:**

The results of one model indicated that greater numbers of traumatic life events experienced by women were associated with raised odds of post-traumatic stress disorder risk (β = 1.48; *p* < 0.001). Another model indicated that exposure to sexual trauma events were associated with greater odds of post-traumatic stress disorder risk (β = 4.09; *p* = 0.02).

**Conclusion:**

Our findings highlight the critical importance of mental health service for females with history of sexual traumatic events for this vulnerable population.

## Introduction

Driven by its economic opportunity and relatively stable democracy, individuals across sub-Saharan African nations seek refuge in South Africa for security and a better livelihood. It is well-recognised that refugees are a vulnerable population who are at increased risk of developing mental health problems, including post-traumatic stress disorder (PTSD).^1^ In addition to the stresses associated with migration itself, and with adjustment to a new country, refugees have been exposed to significant traumatic life events (TLEs), such as physical and sexual violence, imprisonment, famine, war, death of loved ones and lack of security, all of which have a detrimental impact on their mental health.^2^

Systematic reviews of the international literature have consistently shown that PTSD owing to traumatic events exposure is a common challenge among refugees.^2,3^ Limited studies in Somalia and South Africa (about Zimbabwean refugees) also suggest that exposure to traumatic events was significantly associated with having PTSD.^4,5^ Despite the link between exposure to TLEs and the risk of developing PTSD, there is a paucity of studies that examine this association which reflect the diverse nature of migrants within Africa, particularly African female migrants. In the current study, we investigated the association between TLEs on female African refugees (from Zimbabwe, Democratic Republic of the Congo [DRC], Rwanda, Burundi, Uganda, Ghana, Malawi and Mozambique) living in Durban, South Africa, and PTSD risk. Furthermore, it is well established that female refugees/migrants in complex emergencies are prone to being victims of sexual violence.^6^ We therefore hypothesise the effects of sexual trauma events to be a significant risk factor of post-traumatic stress (PTS) among this vulnerable population.

## Research methods and design

### Sampling and data collection

A cross-sectional study was conducted at the Dennis Hurley Centre (DHC) in Durban, South Africa, between July 2013 and April 2014. Dennis Hurley Centre, a faith-based organisation, provides primary health care, paralegal and soup kitchen services for refugees and asylum seekers. While the target study population initially comprised female refugees, it was expanded to include foreign migrants and asylum seekers who did not possess refugee status at the time of the study. It is often difficult to differentiate between true political refugees and economic migrants, as this is a heterogeneous population with complex backgrounds, and formal systems to register refugee status remain poorly developed in South Africa, often being inaccessible. Consecutive DHC women clients who were 18 years or older, non-South African and conversant in English, French or Swahili were invited to participate in the study. Written informed consent was obtained from 157 participants, and study interviews were administered in the language of the participant’s preference. All consent and questionnaire/interview procedures were administered by trained research assistants (nurses) fluent in the noted languages. The participants requiring immediate mental health treatment were referred to local health services.

### Measures

The two main study measures were PTS symptoms and exposure to TLEs. The main dependent variable, PTS symptoms, was measured using the Harvard Trauma Questionnaire (HTQ), which^7^ is a 30-item scale that measures trauma symptom levels during the past week. Reported to have good levels of reliability and validity,^8^ the instrument has been utilised in South African and South Sudanese studies.^4,9^ The responses are based on self-reporting using the same 4-point Likert scale throughout: ‘1 – not at all’, ‘2 – a little bit’, ‘3 – quite a bit’ or ‘4 – extremely’. The study composite score is based on a summation of the first 16 of 30 items, which maps onto the DSM-IV PTS (Cronbach’s alpha = 0.90), with higher scores in HTQ (ranging from 16 to 64), indicating higher severity in PTS. We choose not to dichotomise, given the recent evidence questioning the use of a single cut-off score.^10^

The main covariate, exposure to TLEs, was measured using the Life Events Checklist (LEC) DSM-IV version,^11^ which is reported to have good levels of reliability/validity and has been used in a South African setting.^12^ A list of 18 self-reported TLEs from the LEC, which are traumatic or stressful in nature over the lifetime, was read and presented to the female participants. The events were: natural disasters; fire or explosion; transportation accident; serious accident at home/work; exposure to toxic substance; physical assault; assault with weapon; sexual assault; other unwanted/uncomfortable sexual experience; combat or exposure to a war zone; captivity; life-threatening illness or injury; severe human suffering; sudden violent death; sudden unexpected death of someone close to you; serious injury, harm or death you caused to someone else; and other very stressful events or experiences. For each event, severity of exposure was rated based on the following options: ‘1 – happened to me’, ‘2 – witnessed it’, ‘3 – learned about it’, ‘4 – not sure’, ‘5 – doesn’t apply’. Consistent with a recent study,^13^ total traumatic events were constructed based on the number of ‘1 – happened to me’ responses (ranging from 0 to 18). Exposure to sexual trauma was based on ‘1 – happened to me’ responses from sexual assault (event #8) or other unwanted or uncomfortable sexual experience (event #9). In addition to HTQ and LEC, a sociodemographic questionnaire was administered to obtain information on sex, age, marital status, income, highest educational attainment, and a number of social support sources.

### Analysis

Two analyses were conducted on the data from the three previously stated questionnaires, the first being descriptive statistics methods to summarise the sociodemographic characteristics (from sociodemographic questionnaire), number of traumatic events experienced (from LEC instrument) and severity of PTS (from HTQ instrument). Second, we assessed the relationship between total number of traumatic events and PTS using an adjusted regression model, controlling for age, marital, educational and employment status, nationality, and social support in South Africa. Social support was measured by asking the following questions: How many people do you have here who would give you help and support if you have serious problems? The response choices were ‘none’, ‘1–2’, ‘3–5’, ‘5+’. As most of the responses were either ‘none’ or ‘1–2’, we dichotomised the category to ‘none’ or ‘one or more’. A second regression model was fitted to examine the relationship between exposure to sexual trauma and PTS, adjusted by the same abovementioned covariates.

## Ethical consideration

The University of KwaZulu-Natal Biomedical Research Ethics Committee approved the study.

## Results

### Participant characteristics

The sociodemographic characteristics of the 157 respondents indicated a mean age of 31.45 years (s.d. = 8.85 years), with over half (*n* = 90; 57.32%) being married, approximately two-thirds having at least one source of social support (*n* = 125; 80.13%) and 50.32% (*n* = 78) being unemployed. Participants were from Burundi, DRC, Ghana, Mozambique, Rwanda, Uganda, Malawi and Zimbabwe, with most being from the DRC (*n* = 61; 38.85%) and Zimbabwe (*n* = 70; 44.59%). The median number of TLEs experienced was 4 (IQR = 3), while the mean PTS score was 27.28 (s.d. = 9.05). The three most commonly endorsed lifetime traumatic events were sudden/unexpected death of someone close (*n* = 112; 72.26%), physical assault (*n* = 83; 52.87%) and combat trauma (*n* = 77; 49.68%). The proportion of study participants with exposure to sexual trauma was 24.20% (*n* = 38).

### Association between trauma events and post-traumatic symptomatology

Overall, the results of the adjusted regression model (Table 1) indicate that exposure to a higher number of TLEs was associated with greater odds of PTSD risk (Model 1: β = 1.48; *p* < 0.001). Moreover, exposure to sexual trauma events were associated with greater odds of PTSD risk among women (Model 2: β = 4.09; *p* = 0.02).

**TABLE 1:** Relationship between traumatic events experiences and post-traumatic stress using adjusted regression.

Variable	Model 1		Model 2	
	Adj β	SE	Adj β	SE
**Age category**				
18–20	-	-	-	-
21–24	−0.81	3.33	−0.52	3.48
25–34	1.08	2.98	1.85	3.11
35+	2.27	3.22	3.70	3.34
**Marital status**				
Single	-	-	-	-
Married/living with partner	−1.75	1.75	−1.18	1.83
Divorced/widowed	1.80	2.49	2.58	2.61
**Tertiary education**				
No	−0.46	1.43	0.32	1.50
Yes	-	-	-	-
**Employment status**				
Employed or studying	-	-	-	-
Unemployed/not studying	1.97	1.42	1.68	1.49
**Nationality**				
Zimbabwe	5.38**	1.72	2.84	1.64
Other	−1.62	2.02	−2.75	2.09
DRC Congo	-	-	-	-
**Number of social support**				
≥ 1 supportive persons available	0.58	1.87	−1.04	1.90
None	-	-	-	-
Total number of trauma events	1.48***	0.35	-	-
Exposure to sexual trauma events	-	-	4.09*	1.77

*Source*: Trauma events from Life Events Checklist

* *p*< 0.05,

** *p* < 0.01,

*** *p* < 0.001

## Discussion

This study provides evidence that a greater number of trauma events experienced were associated with higher risk of PTS, this being consistent with previous studies in both refugee^7,14^ and non-refugee populations.^15,16^ Moreover, exposure to sexual trauma among women was associated with higher risk of PTS, as hypothesised, consistent with another previous study.^6^

There are several limitations to our study, the first being that we were only able to translate our instruments into three additional languages, which could not accommodate the diversity of individuals across sub-Saharan Africa. The cultural self-expression issues in reporting stress may have biased our findings. Secondly, we utilised facility-based recruitment, and our study participants may therefore not be representative of the wider refugee and foreign national female migrant population in Durban or South Africa. Lastly, as there was no possible way of verifying the information received, self-report bias cannot be ruled out.

## Conclusion

Notwithstanding these limitations, we believe our study adds important knowledge to the very minimal literature on trauma events and outcomes in African female refugees from diverse backgrounds in other African countries. According to the South African Stress and Health (SASH) study, conditional PTSD risk after TLE exposure in the general population was reported to be 3.5%.^17^ This highlights the need for mental health specialist (and non-mental health clinicians) to be aware of PTSD risk among African refugees and migrants living in South Africa, specifically women, and necessitates the expansion of mental health service particularly for individuals with a history of sexual traumatic events.
